# Translating Research Findings Into an Evidenced-Based Approach to Improve Hospital Practices for Malnutrition

**DOI:** 10.1093/geroni/igaa057.461

**Published:** 2020-12-16

**Authors:** Rachel Deer, Janna Lehe, Colleen James, Erin Hommel

**Affiliations:** The University of Texas Medical Branch at Galveston, Galveston, Texas, United States

## Abstract

Malnutrition is a common problem that often goes unrecognized. In a recent UTMB-OAIC funded pilot, we found 49% of older adults were at risk of malnutrition at hospital admission. Malnutrition is associated with increased length of stay, poorer patient outcomes, and higher risk of mortality. Also, malnutrition severity alters hospital reimbursement rates. In 2018, the UTMB health system recognized the need for institutional guidelines to help identify, diagnose, document, and code mild/moderate/severe malnutrition. At baseline, compared to similar academic medical centers, UTMB ranked in the bottom quartile for malnutrition diagnosis. A multidisciplinary committee formed with physicians, nurses, researchers, dieticians, coding, and information technology. Preliminary data from the pilot study found the Nutritional Risk Screen (NRS) had the best sensitivity, specificity, positive and negative predictive values. The NRS was made more user friendly with scripting/prompts in the electronic medical record (EMR) to improve consistency/compliance among nurses. The Subjective Global Assessment was used in EMR by dieticians to document malnutrition diagnosis. A Best Practice Advisory was created to give physicians the option to easily add malnutrition diagnosis to the problem list. Since “go-live” in February 2019, NRS completion improved from 10.6% to 70.0%. Coding of malnutrition increased from 3.7% to 5.8%. In a 6 month follow-up, 113 patients were found to have direct benefits from the new process, resulting in an estimated financial impact of $945,605. Going forward, we have identified multiple areas of continued education needs to further improve the implementation and uptake of the new screening tool and diagnostic processes.

